# Quantification of titanium and zirconium elements in oral mucosa around healthy dental implants: a case–control pilot study

**DOI:** 10.1007/s00784-023-05099-8

**Published:** 2023-06-04

**Authors:** Norbert Cionca, Julien Meyer, Sophie Michalet, Emmanuel Varesio, Dena Hashim

**Affiliations:** 1 Division of Regenerative Dental Medicine and Periodontology, University Clinics of Dental Medicine, University of Geneva, Geneva, Switzerland; 2 Mass Spectrometry Core Facility (MZ 2.0), Faculty of Sciences, University of Geneva, Geneva, Switzerland

**Keywords:** Titanium 1, Zirconium 2, Spectrophotometry 3, Dental implants, Case–control

## Abstract

**Objectives:**

Metallic particles are detected in different sites of the oral cavity, mainly in patients with peri-implantitis lesions. The aim of this pilot study was to analyze the levels of titanium and zirconium elements in the oral mucosa around healthy implants and to investigate the impact of titanium exogenous contamination on the measurements.

**Materials and Methods:**

Forty-one participants were included in this three-phase study. Two groups of subjects were defined according to presence of titanium or zirconia implants (n: 20) or without any implants nor metallic restorations (n:21). Thirteen patients (n: 5 with zirconia implant; n: 3 with titanium implants; n: 5 control group) took part to the first part designed to optimize and validate the method of detecting titanium (Ti) and zirconium (Zr) elements in the oral mucosa and gingival tissues by the Inductively Coupled Plasma Mass Spectrometry (ICPMS). The second phase compared the levels of Ti and Zr concentrations in patients with implants (n: 12) and without implants (n: 6) who were controlled for their intake of titanium dioxide (TiO2). The last step included ten control subjects without any metallic devices to measure the concentration of Ti and Zr before and after having candies containing TiO2.

**Results:**

In the first phase, concentrations of Ti and Zr were below the limit of detection (LOD) in most cases, 0.18 μg/L and 0.07 μg/L respectively. In the titanium group, two out of three subjects displayed concentrations above the LOD, 0.21 μg/L and 0.66 μg/L. Zr element was only found in patients with zirconia implants. After controlling the intake of TiO2, all concentrations of Ti and Zr were below the limit of quantification (LOQ). Moreover, in patients with no implants, the Ti concentration in gingiva cells was superior for 75% of the samples after having a TiO2 diet.

**Conclusions:**

Zirconium was only found in patients with zirconia implants, whereas titanium was detected in all groups even in subjects with no titanium implants. Zirconium and titanium elements were not detected in patients who were controlled for their intake of food and their use of toothpaste irrespective of the presence of implants or not. For 70% of the patients, the titanium detection was directly influenced by the intake of TiO2 contained candies.

**Clinical relevance:**

When analyzing titanium particles, it is necessary to pay attention to the risk of contamination bias brought by external products. When this parameter was controlled, no titanium particles were detected around clinically healthy implants.

## Introduction

Dentistry is a field in constant evolution. Through multiple innovative therapies and techniques, practitioners can now provide an increasingly widening array of solutions to improve their patients’ oral health care [1]. With implantology at the center of modern practice, titanium implants have repeatedly risen to every challenge [2]. Yet despite their renowned success, the presence of metallic elements in a biological system can induce a variety of physiologic responses disturbing its natural equilibrium [3]. Aseptic loosening was a term first described in orthopedics to characterize the body’s immune reaction to wear debris, resulting in local osteolysis around joint replacements. This phenomenon was also occasionally observed around functional dental implants, which are constantly exposed to microbial challenges in contrast to the sterile environment surrounding hip joints [4]. While the precise cause remains unknown, several hypotheses have been suggested. Recent reports have demonstrated that titanium oral implants could release particles due to mechanical wear, corrosion or in the presence of bacterial biofilm [5]. The production of metal elements could generate multiple local and systemic interactions inducing hypersensitivity, inflammatory reactions, and mutagenicity [6–8]. Studies have also demonstrated higher levels of titanium particles in tissues surrounding peri-implantitis lesions [9–11]. These particles may play a role in the initiation and/or progression of peri-implant diseases [12].

Keeping the concept of « primum non nocere», several studies have focused on the causes of metallic particles’ release as well as their side effects[13]. Different samples have been analyzed; including submucosal plaque, granulation tissue, soft tissue biopsies, hair, saliva and exfoliated oral mucosal cells [9, 14, 15]. Direct observations were made using transmitted light microscopy (LM) or scanning electron microscopy (SEM) associated with energy dispersive X-ray (EDX) spectroscopy [10, 12]. Analytical techniques such as Inductively Coupled Plasma Mass Spectrometry (ICP- MS) or synchrotron radiation X-ray fluorescence spectroscopy (SRXRF), have also been employed in some studies [16–18]. Results were therefore presented in descriptive or semi- quantitative forms depending on the chosen method regardless of the particles’ origin or size. Metallic elements, mostly in the form of titanium oxide (TiO2), are frequently used in the industry. TiO2 powder is found in paintings, cosmetics, dental hygiene products, pharmaceutical additives and even food [19, 20]. Oral, subcutaneous, dermal, intravenous, and respiratory routes could lead to systemic contamination with titanium. Elements can be detected in a metallic, ionic, or oxidized state, and their particles can cluster in different sizes, from nano to macro. Bio-reactivity and immune responses can completely change according to the particles’ configuration [21–23].

Due to the rising concerns surrounding titanium, zirconia ceramic implants have become increasingly popular. Yet their clinical and biomechanical superiority remains to be proven [24]. Moreover, titanium (Ti) and zirconium (Zr) are both exogenous elements widely used in dental biomaterials. Since neither can be naturally found in the human body [15], their presence in a biological milieu indicates some form of external contamination. Several studies have reported the presence of titanium particles in intra- oral tissues as well as distant organs of implant patients [25, 26]. But so far, none have reported on Zr in ceramic implant patients. Only limited data from in vitro and animal studies suggested that both Ti and Zr particles have detrimental effects on the viability and the metabolic activity of osteosarcoma-derived osteoblasts and human gingival fibroblasts but no inflammatory expression from monocytes [27]. An animal study also described that Ti microparticles elicited a higher increase of O_2_^−^ generation in the lung compared to Zr. The authors concluded that the biokinetics of Ti and Zr particles were influenced by the size, shape and/or crystal structure [28]. Yet the variability in sampling techniques, the lack of standardized detection and quantification methods, as well as the differences in data presentation and analysis have impeded direct comparison between studies.

Therefore, the aim of this pilot study was to optimize the level of ICP-MS detection and quantification of Ti and Zr elements in the mucosa of patients with heathy oral implants, and to compare them with a control group. A secondary objective was to assess the presence of titanium in the peri-implant mucosa and in the gingiva of subjects controlled for the exogenous contamination with TiO2.

## Materials and Methods

This is a single-center clinical case–control pilot study divided into three phases. The Ethical Committee of the University Hospitals of Geneva, Geneva, Switzerland, approved the protocol (10–234/Psy 10–031). Research was conducted according to the principles outlined in the Declaration of Helsinki on human medical experimentation.

Subjects were recruited from a pool of patients previously treated with oral implants at the Division of Periodontology as well as from the dental staff of the University Clinics of Dental Medicine (CUMD; Clinique Universitaire de Médecine Dentaire), University of Geneva, Switzerland. All participants were adults 20 years old or over, systemically healthy with no signs of active oral disease.

Exclusion criteria included lack of keratinized tissue (< 2 mm), smoking more than 10 cigarettes per day, addiction to alcohol or other substances, heavily overweight, current major systemic or oral pathologies, poor oral hygiene (full-mouth plaque score (FMPS) > 20%), metallic dental fillings, and subjects not willing to attend regular dental maintenance care and follow up evaluations.

Written informed consent was obtained from all subjects, and each was assigned a number in an ascending order.

**Fig. 1:**
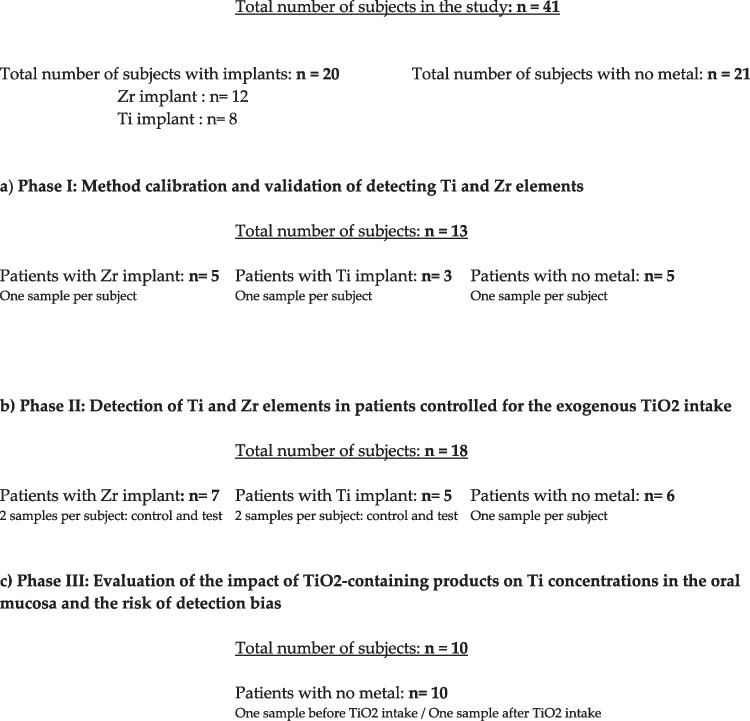
Flowchart of the three-stage pilot study

Forty-one subjects (mean age 43.6 ± 10.2) were enrolled in this three-stage pilot study (Fig. 1). They were recruited at different time points according to the start of the experiment. Two groups were identified: the first included patients with one implant supporting a single crown with a minimum loading period of one year (n = 20; 12 females and 9 males). Twelve patients presented with zirconia implants (Zeramex T® two-piece implant system, Dentalpoint AG, Zürich, Switzerland) and eight with titanium implants (Straumann® Tissue Level, Institute Straumann, Basel, Switzerland). All implants were placed in the posterior region by the same operator (NC) (13 in the maxilla and 7 in the mandible) and healed unsubmerged. All threads were located in pristine bone and no bone augmentation was realized. All implant-supported crowns were made of full-ceramic materials and were either only cemented in the zirconia implant group or cemented/screw-retained in the titanium implant group. Abutments in the titanium implant group were made of titanium alloy. Neighboring teeth were also free of metallic restorations. Clinical and radiological evaluation showed no signs of mucositis or peri-implantitis lesions. The second group included subjects without any implants or metallic restorations (n = 21; 11 females and 10 males).

### Sampling procedure and analysis

Before sampling, all patients were asked to rinse their mouths twice for one minute using a glass of water to remove exfoliated cells. Cytologic specimens were obtained by gently rotating a micro-brush three times (size 2 mm, Microbrush®, Young Microbrush Ireland Ltd. Clogherane, Dungarvan Co. Waterford, Ireland) over the peri-implant buccal mucosa or over the buccal gingival tissues of control teeth. A teflon template was used to assure the reproducibility of sampling. Samples and quality controls (QC) were prepared in 1% PlasmaPure nitric acid (SCP Science, France) and 0.004% hydrofluoric acid for ultra-traces analysis (Otpima, Fisher Chemical, Brunschwig, Suisse), in ultra-pure water (v/v), called the matrix solution. They were then digested at 80 °C for 1 h. The concentrations of the Zr and Ti elements were determined on a 7700 × inductively coupled plasma mass spectrometer (ICP-MS, Agilent Technologies, France) in instrumental triplicates. In addition to the “No Gas” mode, Helium gas (He) was used in a collision/reaction cell (CRC) to reduce and/or remove spectral interferences. Internal standardization (103Rh and 185Re) was used to correct instrumental variability (e.g., ionization efficiency) and to overcome concentration bias due to matrix effects. The metal concentration was in the range of parts per billion (μg/L = ppb). Single external calibration was applied using a standard solution containing 100 μg/L of zirconium and titanium (Techlab, France) in semi-quantitative mode for the first experiment. For the other two experiments, the quantitative mode was used, and an external calibration was applied with eight standard solutions ranging from 0.5 to 250 μg/L of zirconium and titanium in the matrix solution. The linear regression determination coefficients (R2) are superior to 0.9998 for all Ti and Zr isotopes (46Ti, 47Ti, 48Ti, 49Ti, 50Ti, 90Zr, 91Zr, 92Zr, 94Zr and 96Zr) in agreement with the Food and Drug Administration guidelines for Elemental Analysis (FDA: R^2^ ≥ 0.998). The accuracy or recovery (R in %) was evaluated from QC solutions containing digested micro-brushes previously doped with known concentrations in Ti or Zr. The best average recoveries (n = 13) at a theoretical concentration of 2 μg/L were 103% and 105%, respectively for 47Ti no gas and 49Ti no gas and He. Concerning Zr isotopes at the same theoretical concentration, they were equal to 86% and 87% respectively for 90Zr no gas and He, and for 91Zr He. The experimental precision expressed by the relative standard deviation (RSD in %, n = 13) are inferior or equal to 4.5% and 11.3%, for these Ti and Zr isotopes, respectively. The limit of quantification (LOQ) was determined for the quality control solution (QC) accuracy and precision results. The LOQ was defined at the lowest QC concentration with 80% ≤ accuracy ≤ 120% and RSD ≤ 20% according to the guidelines of the FDA. They are equal to 0.5 μg/L and 1 μg/L respectively for 47Ti no gas and He, 49Ti no gas and He, 90Zr He and for 90Zr no gas, 91Zr no gas and He. The limit of detection (LOD) was calculated as 3 standard deviations added to the mean value of the background signal of 3 to 5 blank matrix intensities (digested Microbrush in the matrix solution), which would be converted into analyte concentration (recommendation by FDA), as follow: LOD = IA + 3 SD with SD = Standard Deviation and IA = Intensity Average.

### Clinical protocols

#### Phase I

The aim of the first phase was to calibrate and validate the method of detecting Ti and Zr elements in the oral mucosa and gingival tissues. A semi-quantitative measurement mode was selected. Thirteen patients were recruited in October 2017. Cytologic specimens were obtained from the peri-implant mucosa of five patients with zirconia implants, three patients with titanium implants, and from the gingiva of five individuals without any implants or metallic restorations. Three blank micro-brushes were analyzed to exclude any contaminants and artifacts from the material itself.

#### Phase II

Eighteen participants were recruited in February 2018. Six patients did not have any implants, seven presented with single zirconia implants and five with single titanium implants. After validation and optimization of the method, the second phase aimed to include a control site (the contra-lateral tooth) in the implant group. Participants were asked to refrain from using toothpastes or consuming candies 12 h before sampling. Two cytological samples were harvested from each implant patient as previously described: one from the peri-implant mucosa and one from the gingival tissues of the contra-lateral tooth. Only one sample was taken from control patients without implants. Thirty specimens were analyzed.

#### Phase III

The third stage aimed to evaluate the impact of TiO2-containing products on Ti concentrations in the oral mucosa, and the risk of detection bias when using the ICP-MS method. Ten subjects without implants or metallic fillings were included in November 2019. They were asked to neither have candy nor brush their teeth with a toothpaste 12 h before sampling. Once the first sample was harvested, the subjects were given a candy to masticate for two minutes before a second sample was collected.

#### Statistical analysis

The concentration levels of Ti and Zr were measured, and the data is presented in a descriptive manner. As a pilot study, power analysis was not required.

## Results

### Phase I

Sixteen samples were analyzed by ICP-MS and concentrations of Ti and Zr were measured in a semi-quantitative mode. The detection limits were established at 0.18 μg/L and 0.07 μg/L (ppb), for Ti and Zr respectively. None of the control samples showed traces of either element.

Table 1 details the preliminary findings. Ti was detected in one subject in the zirconia group and one control subject without implants, with concentrations of 2.3 μg/L and 0.31 μg/L, respectively.

**Table 1:** Ti and Zr concentrations measured by ICP-MS in semi-quantitative mode in blank samples (N1 to N3), in samples from patients without implants (S1 to S5), with a titanium implant (T1 to T5) and with a zirconia implant (Z1 to Z5)

Sample N°	^47^Ti [He]		^90^Zr [He]	
	Measured concentration (ppb)	Signal intensity (cps)	Measured concentration (ppb)	Signal intensity (cps)
N1	n.a	67	n.a	483
N2	n.a	0	n.a	467
N3	n.a	33	n.a	300
S1	0.31	150	< LOD**	333
S2	< LOD*	17	< LOD**	383
S3	< LOD*	0	< LOD**	217
S4	< LOD*	0	< LOD**	350
S5	< LOD*	50	< LOD**	517
Z1	< LOD*	33	0.08	834
Z2	2.3	1109	0.07	734
Z3	< LOD*	33	< LOD**	400
Z4	< LOD*	33	0.24	2601
Z5	< LOD*	67	0.07	800
T1	0.66	317	< LOD**	667
T2	< LOD*	0	< LOD**	167
T3	0.21	100	< LOD**	533

Limits of detection (LOD): * for 47Ti [He] = 0.18 ppb, ** for 90Zr [He] = 0.07 ppb

In the titanium group, two out of three subjects displayed Ti concentrations exceeding the LOD, 0.21 μg/L and 0.66 μg/L.

In the zirconia group, 4 out of 5 subjects showed presence of Zr element. The measured concentrations were 0.07 μg/L for two, 0.08 μg/L for one and 0.24 μg/L for one. Zr element was only found in patients with zirconia implants.

### Phase II

Thirty samples were available for the second stage of analysis.

In patients with no implants, concentrations of both Ti and Zr elements were below the LOD (Table 2a).

**Table 2:** Ti and Zr concentrations measured by ICP-MS in semi-quantitative mode in blank samples in 30 samples a) from patients without implants (D1 to D6), b) from patients with one titanium implant (Ti1-1 to Ti5-1) and the contra-lateral tooth (Ti1-2 to Ti5-2) and c) from patients with one zirconia implant (Zr1-1 to Zr7-1) and the contra-lateral tooth (Zr1-2 to Zr7-2)

a) Patients without implants															
N°	^47^Ti (ppb)		^90^Zr (ppb)			^91^Zr (ppb)			^92^Zr (ppb)	^94^Zr (ppb)			^96^Zr (ppb)		
	[No gas]	[He]	[No gas]	[He]	[HE He]	[No gas]	[He]	[HE He]	[No gas]	[No gas]	[He]	[HE He]	[No gas]	[He]	[HE He]
D1	< LOD	< LOD	< LOD	< LOD	< LOD	< LOD	< LOD	< LOD	< LOD	< LOD	< LOD	< LOD	< LOD	< LOD	< LOD
D2	< LOD	< LOD	< LOD	< LOD	< LOD	< LOD	< LOD	< LOD	< LOD	< LOD	< LOD	< LOD	< LOD	< LOD	< LOD
D3	< LOD	< LOD	< LOD	< LOD	< LOD	< LOD	< LOD	< LOD	< LOD	< LOD	< LOD	< LOD	< LOD	< LOD	< LOD
D4	< LOD	< LOD	< LOD	< LOD	< LOD	< LOD	< LOD	< LOD	< LOD	< LOD	< LOD	< LOD	< LOD	< LOD	< LOD
D5	< LOD	< LOD	**0,13**	**0,14**	**0,13**	**0,13**	**0,13**	**0,13**	**0,14**	**0,14**	**0,15**	**0,15**	< LOD	< LOD	< LOD
D6	< LOD	< LOD	**0,59**	**0,59**	**0,58**	**0,59**	**0,59**	**0,57**	**0,60**	**0,61**	**0,63**	**0,61**	**0,65**	< LOD	< LOD
b) Patients with one titanium implant															
N°	^47^Ti (ppb)		^90^Zr (ppb)			^91^Zr (ppb)			^92^Zr (ppb)	^94^Zr (ppb)			^96^Zr (ppb)		
	[No gas]	[He]	[No gas]	[He]	[HE He]	[No gas]	[He]	[HE He]	[No gas]	[No gas]	[He]	[HE He]	[No gas]	[He]	[HE He]
Ti1-1	< LOD	< LOD	< LOD	< LOD	< LOD	< LOD	< LOD	< LOD	< LOD	< LOD	< LOD	< LOD	< LOD	< LOD	< LOD
Ti2-1	< LOD	< LOD	< LOD	< LOD	< LOD	< LOD	< LOD	< LOD	< LOD	< LOD	< LOD	< LOD	< LOD	< LOD	< LOD
Ti3-1	**0,11**	< LOD	< LOD	< LOD	< LOD	< LOD	< LOD	< LOD	< LOD	< LOD	< LOD	< LOD	< LOD	< LOD	< LOD
Ti4-1	< LOD	< LOD	< LOD	< LOD	< LOD	< LOD	< LOD	< LOD	< LOD	< LOD	< LOD	< LOD	< LOD	< LOD	< LOD
Ti5-1	< LOD	< LOD	< LOD	< LOD	< LOD	< LOD	< LOD	< LOD	< LOD	< LOD	< LOD	< LOD	< LOD	< LOD	< LOD
Ti1-2	< LOD	< LOD	< LOD	< LOD	< LOD	< LOD	< LOD	< LOD	< LOD	< LOD	< LOD	< LOD	< LOD	< LOD	< LOD
Ti2-2	< LOD	< LOD	< LOD	< LOD	< LOD	< LOD	< LOD	< LOD	< LOD	< LOD	< LOD	< LOD	< LOD	< LOD	< LOD
Ti3-2	**0,14**	< LOD	< LOD	< LOD	< LOD	< LOD	< LOD	< LOD	< LOD	< LOD	< LOD	< LOD	< LOD	< LOD	< LOD
Ti4-2	< LOD	< LOD	< LOD	< LOD	< LOD	< LOD	< LOD	< LOD	< LOD	< LOD	< LOD	< LOD	< LOD	< LOD	< LOD
Ti5-2	< LOD	< LOD	< LOD	< LOD	< LOD	< LOD	< LOD	< LOD	< LOD	< LOD	< LOD	< LOD	< LOD	< LOD	< LOD
c) Patients with one zirconia implant															
N°	^47^Ti (ppb)		^90^Zr (ppb)			^91^Zr (ppb)			^92^Zr (ppb)	^94^Zr (ppb)			^96^Zr (ppb)		
	[No gas]	[He]	[No gas]	[He]	[HE He]	[No gas]	[He]	[HE He]	[No gas]	[No gas]	[He]	[HE He]	[No gas]	[He]	[HE He]
Zr1-1	< LOD	< LOD	< LOD	< LOD	< LOD	< LOD	< LOD	< LOD	< LOD	< LOD	< LOD	< LOD	< LOD	< LOD	< LOD
Zr2-1	< LOD	< LOD	< LOD	< LOD	< LOD	< LOD	< LOD	< LOD	< LOD	< LOD	< LOD	< LOD	< LOD	< LOD	< LOD
Zr3-1	< LOD	< LOD	**0,07**	**0,07**	**0,07**	**0,07**	**0,07**	**0,07**	< LOD	< LOD	< LOD	< LOD	< LOD	< LOD	< LOD
Zr4-1	< LOD	< LOD	< LOD	< LOD	< LOD	< LOD	< LOD	< LOD	< LOD	< LOD	< LOD	< LOD	< LOD	< LOD	< LOD
Zr5-1	< LOD	< LOD	< LOD	< LOD	< LOD	< LOD	< LOD	< LOD	< LOD	< LOD	< LOD	< LOD	< LOD	< LOD	< LOD
Zr6-1	< LOD	< LOD	< LOD	< LOD	< LOD	< LOD	< LOD	< LOD	< LOD	< LOD	< LOD	< LOD	< LOD	< LOD	< LOD
Zr7-1	**0,15**	< LOD	< LOD	< LOD	< LOD	< LOD	< LOD	< LOD	< LOD	< LOD	< LOD	< LOD	< LOD	< LOD	< LOD
Zr1-2	< LOD	< LOD	< LOD	< LOD	< LOD	< LOD	< LOD	< LOD	< LOD	< LOD	< LOD	< LOD	< LOD	< LOD	< LOD
Zr2-2	< LOD	< LOD	< LOD	< LOD	< LOD	< LOD	< LOD	< LOD	< LOD	< LOD	< LOD	< LOD	< LOD	< LOD	< LOD
Zr3-2	< LOD	< LOD	< LOD	< LOD	< LOD	< LOD	< LOD	< LOD	< LOD	< LOD	< LOD	< LOD	< LOD	< LOD	< LOD
Zr4-2	< LOD	< LOD	**0,14**	**0,14**	**0,14**	**0,15**	**0,15**	**0,12**	**0,15**	**0,15**	**0,15**	**0,15**	< LOD	< LOD	< LOD
Zr5-2	< LOD	< LOD	< LOD	< LOD	< LOD	< LOD	< LOD	< LOD	< LOD	< LOD	< LOD	< LOD	< LOD	< LOD	< LOD
Zr6-2	< LOD	< LOD	< LOD	< LOD	< LOD	< LOD	< LOD	< LOD	< LOD	< LOD	< LOD	< LOD	< LOD	< LOD	< LOD
Zr7-2	< LOD	< LOD	< LOD	< LOD	< LOD	< LOD	< LOD	< LOD	< LOD	< LOD	< LOD	< LOD	< LOD	< LOD	< LOD

In the titanium group, only one sample taken from the peri-implant mucosa presented with detectable Ti levels of 0.11 ppb. Neither Ti nor Zr elements were detected in the remaining 4 samples harvested adjacent to implant sites (Table 2b). On the other hand, samples from the contra-lateral teeth of two subjects showed detectable Ti levels of 0.14 and 0.33 ppb. However, none of the detected Ti concentrations reached the LOQ level of 0.5 ppb.

In the zirconium group, around implants, concentrations of Ti and Zr were below the LOD except for two patients where Zr was detected at levels of 0.07 ppb and 0.15 ppb (Table 2 c). Samples around contra-lateral teeth were all negative except for one where a concentration of 0.12 ppb was detected. Again, all three positive Zr samples remained below the LOQ of 2 ppb.

### Phase III

Ti and Zr concentrations were measured in eighteen out of the 20 initially harvested samples. A laboratory error led to the exclusion of 2 samples taken from one subject.

Zr was detected in two samples which showed lower concentrations than that of the LOQ.

**Fig. 2:**
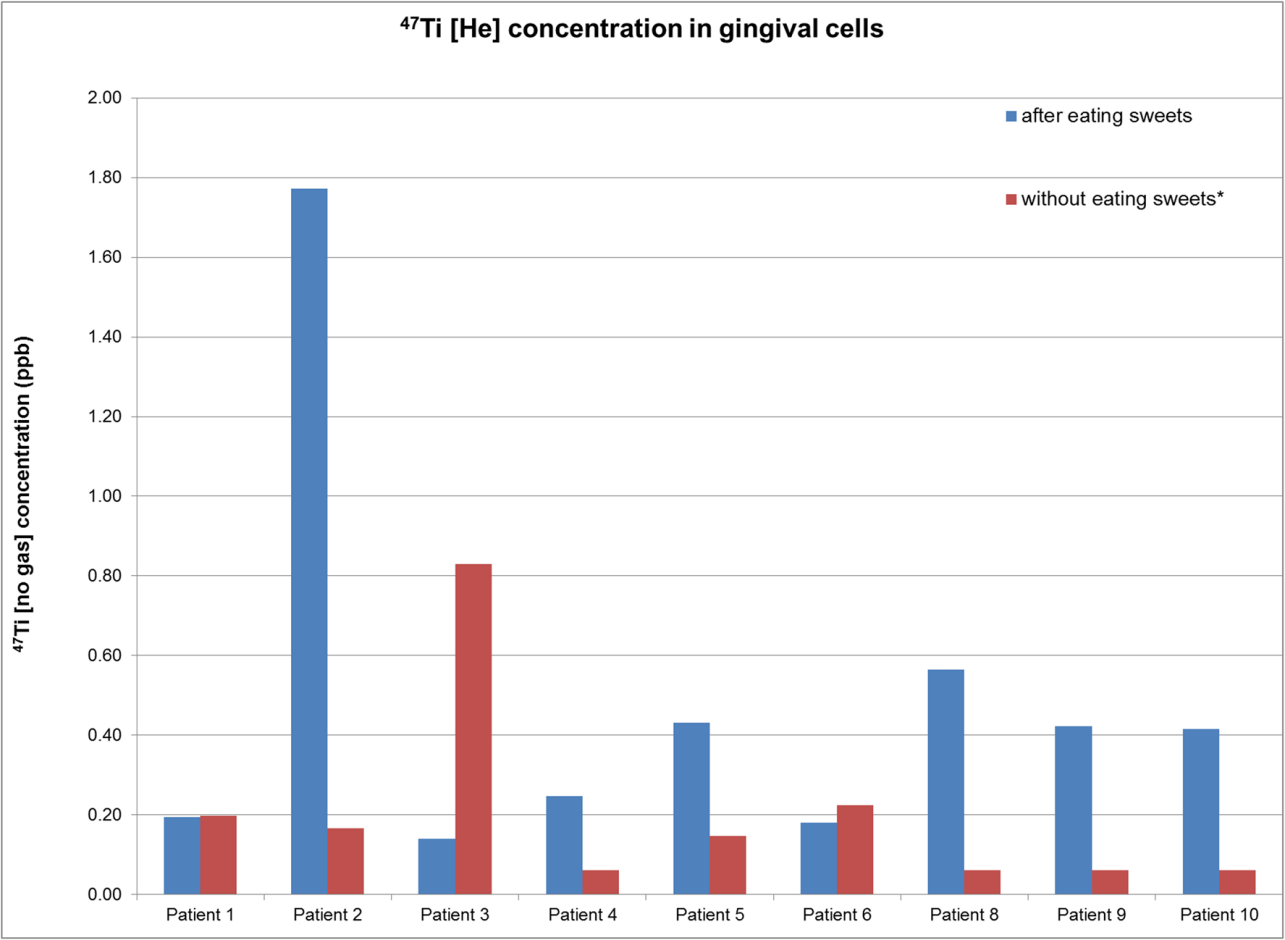
Comparison between the 47Ti [He] concentrations after and before eating sweets, for nine healthy patients

**Fig. 3:**
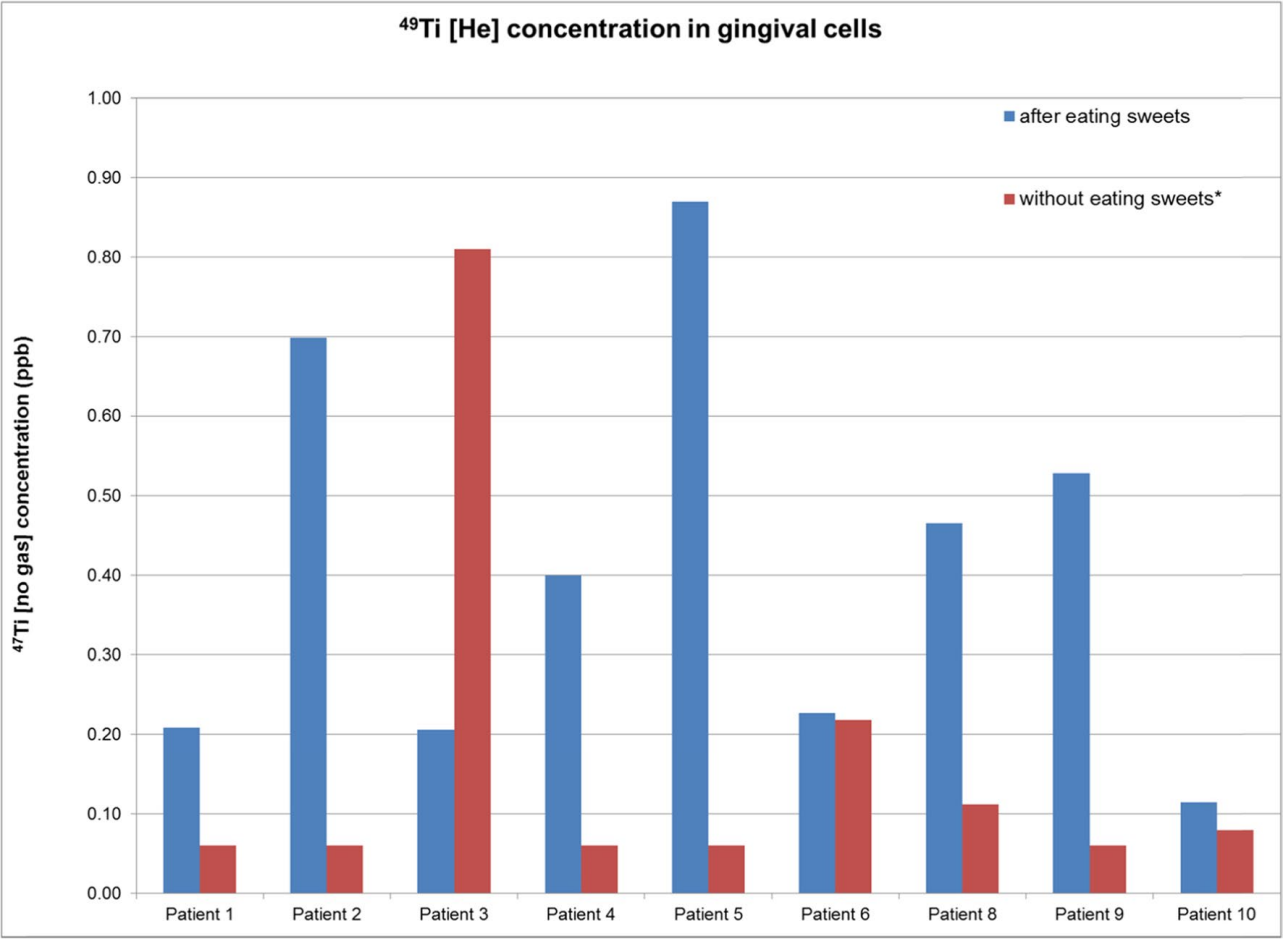
Comparison between the 49Ti [He] concentrations after and before eating sweets, for nine healthy patients

Concerning the presence of Ti elements, six out of nine subjects (66.7%) expressed marked increase in Ti concentrations after eating TiO2-containing candies, whereas two patients did not demonstrate any substantial increase (Table 3). Ti concentration in gingival cells was superior for 75% of the samples after having a TiO2 diet. On the contrary, one subject (patient 3) demonstrated remarkably lower Ti levels after eating the candies (Figs. 2 and 3). A dysfunction in sample manipulation could be the origin of this anomaly.

**Table 3:** Ti concentration in gingival cells before and after eating sweets for each patient are compared in Table 3, by the way of the relative percent difference. When one of these two concentrations is lower than the LOD, no value can be calculated, and a cross is written in the cell and the following color code is applied: • Green if the Ti concentration in gingival cells after eating sweets is superior to those before eating sweets. • Red if the Ti concentration in gingival cell after eating sweets is inferior to those before eating sweets. • Black if there is no significant difference between both Ti concentrations (RPD ≤ 20%)

Patient Name	Relative Percent Difference								
	^47^Ti [No Gas]	^47^Ti [He]	^47^Ti [HEHe]	^49^Ti [No Gas]	^49^Ti [He]	^49^Ti [HEHe]	^50^Ti [No Gas]	^50^Ti [He]	^50^Ti [HEHe]
Patient 1	X	-2%	X	X	X	X	68%	X	X
Patient 2	X	166%	X	X	X	150%	X	X	X
Patient 3	X	-142%	-124%	X	-119%	-164%	X	-176%	-62%
Patient 4	X	X	X	X	X	X	X	X	X
Patient 5	51%	98%	-2%	X	X	X	-111%	**-80%**	X
Patient 6	-54%	-22%	X	-12%	4%	X	-33%	-8%	X
Patient 8	X	X	X	X	122%	X	116%	114%	141%
Patient 9	X	X	X	X	X	X	X	90%	X
Patient 10	X	X	X	X	37%	41%	X	X	X

## Discussion

This pilot study has confirmed cytological sampling as a valid method to diagnose the presence of Ti and Zr elements in the oral mucosa. It has also demonstrated the ICP-MS’s ability to detect these elements at thresholds as low as 0.18 μg/L and 0.07 μg/L (ppb) for Ti and Zr respectively. Zirconium was only found in patients with zirconia implants, whereas titanium was detected even in subjects without any titanium implants or metallic restorations. In most cases, Ti and Zr concentrations were below their levels of detection. The number of participants to this first phase could be a limitation, however the aim at that point was the calibration and validation of the ICP-MS. Another group investigated the presence of Ti in the mucosa of healthy implants and those with peri-implantitis lesions [29]. Titanium concentration was higher in the pooled samples harvested from the peri-implantitis group compared to the healthy one, 2.44 ppb vs 0.88 ppb respectively. Samples taken from the mucosa of contra-lateral teeth, however, did not present with any Ti particles. Corrosion, due to the acidic environment generated by bacteria, could explain the release of these particles into the inflamed peri-implant tissues. Nevertheless, one should keep in mind that measurements were obtained after having pooled the samples together in each group. In our first experiment, one patient presented with a Ti concentration of 2.3 ppb even without having a titanium implant. A detailed interview revealed the patient as a heavy consumer of chewing-gums. Thus, the risk of detection bias remains high due to the cumulative and persistent contamination of TiO2 by different exogenous sources. Nevertheless, and despite the small number of samples, it is noteworthy that zirconia elements were only detected around zirconia implants. This was in contrast to the results of another group of authors who analyzed 36 biopsies from 31 patients with peri-implantitis affecting Ti fixtures [17]. Zr particles were detected in 19 samples with mean proportions ranging between 0.1% to 5.8% per sample. This phenomenon was partially explained by the content of the dental cement where zirconia dioxide was used as a radiopaque additive. Another explanation was the wear induced by cyclic loading at the interface between titanium implants and zirconia abutments [30]. An in vitro study demonstrated zirconia and titanium particles could initiate an immune response mediated by an increase expression of mRNA for Toll-like receptors (TLRs) 2, 3, 4 and 9, and their adaptors MyD88, TRIF and NF-κB,in as well as cytokines TNF-α, IL-1β and IL-6, finally leading to osteolysis, inflammatory hyperalgesia and edema [31]. Interestingly, the intensity of the immunological reactions was lower when macrophages were challenged by zirconia particles, thus suggesting a lower level of bioactivity when compared to Ti.

Exfoliative cytology was not the only method used to highlight and quantify the number of titanium particles in the literature. Plaque samples and biopsies have also been tested in other experiments. A clinical study measured the level of titanium particles in submucosal plaque samples [11] harvested from 30 patients with 20 healthy implants and 20 implants with peri-implantitis. Adjusted titanium levels (nanogram per microliter) were significantly higher in the peri-implantitis group compared to the healthy one, 0.85 ± 2.47 vs 0.07 ± 0.19 respectively. In other words, a significant association between titanium dissolution and peri-implantitis’s implication in corrosion was stipulated.

Titanium elements were examined in different punch biopsies taken from the oral mucosa over submerged implants [9, 16, 32]. All studies established the presence of titanium particles incorporated in the soft tissues. In one study, 41% of the samples (63 out of 153) displayed titanium particles [9]. Two other experiments [16, 32] found Ti elements in more than 95% of their samples. Both studies utilized laser ablation-inductively coupled plasma-mass spectrometry technology but included smaller sample sizes; 6 and 30 patients respectively. Interestingly, control samples also tested positive for titanium. Ti concentration values did not significantly vary between the test (50.4 μg/g ± 23.5 μg/g) and the control groups (37.1 μg/g ± 1.0 μg/g). However, Ti level variations were high between patients with oral implants. Moreover, the particles’ localization within the tissues was neither homogenous nor uniform in distribution. Most particles were detected in the epithelial layers and at the border of the connective tissue in contact with the cover screw [9, 16]. Again, the origin of these particles must be analyzed with caution. In addition to corrosion, mechanical wear during implant insertion or abutment fixation could contribute to Ti particles’ release into the peri-implant tissues.

In order to understand the impact of TiO2 in alimentary industry on Ti detection levels, all patients were controlled for their TiO2 intake before the sampling in the second experiment. All measurements realized around natural teeth, titanium and zirconia implants were below the limits of quantification. This therefore indicates the extra-oral origin of the majority of Ti particles in a non-inflammatory environment. This is further confirmed by a previous study [33]. The later study could not find a significant difference in 47Ti concentrations in patients presenting metal-porcelain fixed crowns on dental implants compared to those without any restorative treatment. This contradicted the results described by another group, where the levels of Ti elements was higher in the exfoliated oral mucosal cells harvested around titanium implants compared to natural teeth [34]. This difference was calculated from two average concentrations of 47Ti obtained from either the implant or the control groups (2.42 μg/L ± 5.05 μg/L vs 0.46 μg/L ± 1.13 μg/L, respectively). However, since the standard deviations were higher than the means, this difference should be relativiz ed.

Since all Ti and Zr levels were below the LOD in our second experiment, a counting method of the harvested mucosal cells and a reduction in the volume of the digestive solution could improve the precision of the measures. In our study, the number of measured mucosal cells was 2.54 × 104 per mL with a 48% coefficient of variation (Countless II FL Automated Cell Counter®, Thermo Fisher Scientific). This level of quantification is comparable to other studies [35, 36].

In patients without any metallic restorations or implants, the last experiment clearly showed that the ICP-Ms method could detect the presence of TiO2 in a healthy gingiva. It was also apparent that the majority of the detected Ti elements were derived from an external alimentary source. However, a certain number of measures were comprised between the LOD and the LOQ for patients having eaten sweets before the analysis. Different isotopes were probed: 47Ti, 49Ti, 50Ti, 90Zr, 91Zr, 92Zr, 94Zr and 96Zr. The 46Ti and 48Ti isotopes were not investigated because of their interferences with 46Ca and 48Ca which form parts of the micro-brush composition. In the present analytical batch, the two most accurate and sensitive conditions were found for isotopes 47 and 49 in a helium mode. Therefore, the sensitivity of the analytical workflow could further be improved either by the optimization of the sample preparation (reduction of the digestion volume, use of another sampling tool in polytetrafluoroethylene) or by the optimization of the analytical method (i.e. counting the number of cells).

Another point to consider is that although the dosage was only realized in a semi-quantitative mode for phase I, 90Zr quantification errors were twice higher than those of Ti. Nevertheless, the stability of the signal of the internal standardization (103Rh) was consistent with the FDA recommendations. Other tests were conducted to examine the presence of some interfering elements (Hf, Ge, Se, Cr, Fe et V) disturbing the quantification of 90Zr. But since these elements did not enter the composition of the multi-element solution, they could not explain the interference in quantification. In our study, the method of Ti and Zr quantification using ICP-MS was confirmed in a linearity range of 0.5 to 250 ppb for Ti and of 1 to 250 ppb for Zr. Limits of detection were in the range of 0.11 for 47Ti (no gas) and 0.15 ppb for 47Ti (He). Concerning Zr elements, limits of detection were between 0.04 and 0.91 ppb depending on the Zr isotope and CRC mode.

In addition to their concentration levels, the particles’ characteristics play a major role on both local and systemic biological responses to a Ti stimulus [37]. Titanium particles can activate osteoclasts by increasing the expression of receptor activator of nuclear factor ĸB ligand (RANKL) [38] and stimulating the production of TNF-α, IL-1β, IL-6 and IL-8 by macrophages and lymphocytes [31, 39]. Furthermore, a specific cascade was described where titanium particles played the role of a secondary stimulus in the release of IL-1β from macrophages activated by bacterial lipopolysaccharides [10]. Ti particles’ size, shape, and their level and pattern of aggregation could modulate these immune responses [20, 21, 40]. Several experiments have demonstrated that nanoparticles showed higher cytotoxicity resulting in greater secretion of pro-inflammatory mediators compared to microparticles [22]. Due to technical limitations, clinical studies scarcely identified the size of the particles detected in their biopsies. Also, it is important to remember that the ICP-MS can detect the presence of titanium but discrimination between the different states; such as oxidized, metallic and ionic, remains difficult and sensitive [41]. A group of authors investigated the titanium concentration in granulation tissues harvested from thirteen patients with peri-implantitis and eleven with periodontitis [10]. A higher concentration of titanium was found in the peri-implantitis group compared to the periodontitis one (98.7 ± 85.6 μg/g vs 1.2 ± 0.9 μg/g, respectively) but the elemental composition analysis using the EDX only showed Ti signals in the peri-implantitis samples. The size of the particles varied from macro to microparticles without any nanoparticles. However, most implants were scaled beforehand which could impact titanium concentrations in the harvested samples. Despite treatment, all patients lost at least one implant. This was attributed to the combination of bacterial biofilm and Ti particles which could enhance and sustain inflammation and bone resorption. It must be stressed all participants in this three-phase study presented healthy implants without any signs of inflammation.

Presence of titanium particles has become common and well described in layers closed the implant surface and in peri-implantitis cases [13]. Mechanical wear, biocorrosion and tribocorrosion could explain the release of titanium particles and degradation products of titanium in the oral environment [5]. Several in vitro experiments have demonstrated effects of titanium particles on bone and soft tissues [42–44]. Aseptic loosening of the implant could be one of the consequences of the linear osteolysis induced by the inflammatory effects of titanium particles on macrophages and lymphocytes [45]. However, this complex interaction between different actors remains difficult to study clinically.

Further investigations need to compare the levels of Ti and Zr particles in patients with peri-implantitis to understand the consequences of their presence in the oral cavity. Care must be taken when analyzing future results to exclude any contamination from exogenous TiO2 which could influence levels of titanium in the soft tissues.

## Conclusions

Zirconium was only found in patients with zirconia implants, whereas titanium was detected in all groups even in subjects with no titanium implants. Zirconium and titanium elements were not detected in patients who were controlled for their intake of food and their use of toothpaste irrespective of the presence of implants or not. For 70% of the patients, the titanium detection was directly influenced by the intake of TiO2 contained candies.

## Data Availability

I Data available on request due to restrictions eg privacy or ethical. The data presented in this study are available on request from the corresponding author.
